# Comparison of accusations against physicians and the practice of defensive medicine between surgical and non-surgical specialties

**DOI:** 10.1371/journal.pone.0343807

**Published:** 2026-03-05

**Authors:** Ayat Mahmoud Tawfik, Safaa ElZoghby, Noura Mahmoud Elsherbiny, Marwa Rashad Salem

**Affiliations:** 1 Public Health and Community Medicine Department, Faculty of Medicine, Port-Said University, Port-Said, Egypt; 2 Family Medicine Department, Faculty of Medicine, Suez Canal University, Ismailia, Egypt; 3 Public Health and Community Medicine Department, Faculty of Medicine, Suez Canal University, Ismailia, Egypt; 4 Public Health and Community Medicine Department, Faculty of Medicine, Cairo University, Giza, Egypt; Mediterranean University of Reggio Calabria: Universita degli Studi Mediterranea di Reggio Calabria, ITALY

## Abstract

**Background:**

Defensive medicine has two forms: positive (assurance behavior) or negative (avoidance behavior), depending on the clinical situation. Defensive medicine minimizes the risk of litigation and tends to vary between surgical and non-surgical specialties due to the nature of the risks involved and the potential for litigation. This study aimed to investigate the prevalence and patterns of defensive medicine practice among Egyptian physicians, compare surgical versus non-surgical specialties, and examine their correlation with medico-legal complaints and occupational determinants.

**Methods:**

This cross-sectional study was conducted among physicians from surgical and non-surgical specialties working in different Egyptian hospitals. A self-administered online questionnaire was distributed using the snowball sampling technique. The Defensive Medicine Behavior Scale (DMBS) was used to assess the practice of defensive medicine.

**Results:**

A sample of 210 physicians with a mean age of 39 ± 7 years was included; 51.4% held the highest qualification of M.D. or Ph.D., with an equal sex distribution (1:1). There was a high level of defensive medicine practice in both surgical and non-surgical specialties: 41.7% and 39.5%, respectively. However, the difference between the two groups was not statistically significant (P-value >0.05). Regression analysis showed that working at university hospitals and having workplace insurance coverage for medico-legal claims were associated with fewer positive defensive medicine practices. Conversely, concerns about the financial implications of medico-legal claims and negative reactions from patients or families were associated with a greater prevalence of positive defensive medicine practices.

**Conclusion:**

Despite the high prevalence of defensive medicine practices, no statistically significant differences were observed between the surgical and non-surgical groups regarding overall engagement in defensive medicine.

## Introduction

Malpractice claims against physicians, whether justified or not, have increased in recent years [1]. Such legal cases have a devastating effect on physicians’ thoughts, emotions, and behaviors, particularly when claims are unfounded [2,3]. Furthermore, legal proceedings consume considerable time, adding to the burden of physicians already overburdened with professional responsibilities and negatively affecting their social lives [4]. Additionally, litigation may cause physician distress, depression, and burnout, which negatively impacts their interactions and communication with patients and their families, as demonstrated in many studies [5–7].

Defensive medicine (DM), defined as medical practices primarily designed to minimize the risk of malpractice litigation rather than to enhance patient care, is a well-documented response to this litigious climate [8]. Its prevalence, typically measured through physicians’ surveys and healthcare utilization studies, is widespread across numerous specialties [9]. The practice is influenced by a complex interplay of factors including the legal environment, clinical uncertainty, and physician risk management. DM represents a significant burden on healthcare systems, leading to resource waste, potential patient harm from overdiagnosis, and moral distress among health professionals [10].

A survey of physicians in six lawsuit-prone specialties (emergency medicine, general surgery, orthopedic surgery, neurosurgery, obstetrics/gynecology, and radiology) showed that nearly 93% had altered their practice due to the fear of impending or pending lawsuits. The most common change reported was the adoption of DM, a practice that often deviates from or contradicts established standards of appropriate medical care [11].

DM can manifest as either positive (assurance behavior) or negative (avoidance behavior). The positive form involves undertaking excessive diagnostic tests and invasive investigations, prescribing treatment without a clear medical need, and hospitalizing patients unnecessarily. Conversely, the negative form entails failing to perform high-risk procedures that would benefit patients, thereby potentially denying them necessary treatment or hospitalization [12].

DM is performed primarily to mitigate litigation risk [13]. While the denial of necessary interventions is unethical and impedes accurate diagnosis and treatment, positive DM also threatens patient safety [14]. Such practices can expose patients to unnecessary risks, such as radiation exposure or invasive procedures, and generate additional costs as well as prolonged hospital stays [15].

The practice of DM tends to vary between surgical and non-surgical specialties, influenced by the inherent nature of clinical risks and the specific litigation exposure associated with each field [16]. The invasive nature of surgical procedures and the potential for physical harm often place surgeons in a defensive stance, while non-surgical professionals may resort to over-testing or excessive documentation as a means of self-protection. Nonetheless, both strategies can lead to increased healthcare spending and unnecessary patient exposure to medical procedures [17].

Whereas there is no formal economic estimate of the cost of DM in Egypt, despite its widespread practice, in the United States, annual costs are estimated to range from $50 to $100 billion. At the patient level, this adds approximately $226 per hospital stay, representing 13% of the average stay cost of $1,695 [18,19]. Similarly, an Italian study calculated that DM accounted for up to 10% of total annual national health expenditure [20].

Recently, in 2025, Egypt enacted Medical Responsibility Law No. 13/2025, which establishes rules to improve physician accountability by distinguishing unavoidable complications from true malpractice. While intended to enhance patient safety, the law’s unclear definition of malpractice may prompt physicians to practice more cautiously to avoid punishment [21]. Previous research has documented widespread DM among Egyptian physicians [22–26], but without comparing the incidence of accusations and defensive practices between surgical and non-surgical specialties.

This study aimed to investigate the prevalence and patterns of DM among Egyptian physicians, compare surgical versus non-surgical specialties, and examine their correlation with medico-legal complaints and occupational determinants. It also sought to identify physicians’ concerns regarding potential medico-legal accusations, thereby providing insights into the institutional and professional determinants of DM within Egypt’s healthcare context.

## Materials and methods

### Study design and participants

This cross-sectional study was conducted over a three-month period from February to April 2024. The inclusion criteria were currently licensed physicians of any age and from either sex, working in surgical or non-surgical specialties at the following types of hospitals: university, military, health insurance organization, Ministry of Health, private sector, or Primary Health Care Units (PHCUs). Exclusion criteria comprised physicians who were retired or temporarily not practicing at the time of the study, including those on leave, interns, and medical students. Specialties were classified as either surgical or non-surgical to distinguish between physicians whose practice is primarily high-procedure and higher-risk (surgical) and those whose work is mainly non-operative (non-surgical), thereby capturing major contrasts in risk exposure. We acknowledge, however, that some overlap inevitably exists [14].

Egypt’s health sector is characterized by a dual structure comprising a resource-constrained public sector, which serves the majority of the population, and an evolving private sector that typically offers better facilities and shorter waiting times. The Universal Health Insurance system is being implemented progressively across Egypt, with the goal of covering all governorates by 2032. The implementation will occur over six phases, each focusing on a distinct geographic area (i.e., a cluster of governorates).

Medico-legally, medical practice is governed by the Egyptian Physicians’ Syndicate Law and the Penal Code, which establish the paradigms for professional conduct and medical negligence. However, the prevailing malpractice lawsuit system is generally regarded as time-consuming and complex; this can negatively affect clinical decision-making and the reporting of complications.

### Sample size and design

The sample size was determined using the CDC’s Epi-Info software calculator, with a 95% confidence level and an alpha error of 5%, based on differences in DM practices between specialties reported in a previous Egyptian study [21]. The calculated sample size was 210 physicians. Data were collected using a self-administered online questionnaire and the snowball sampling method. The process began by distributing the survey to known contacts, who were then asked to share it with their colleagues. This technique was appropriate for investigating this sensitive topic, as physicians may be hesitant to openly discuss such issues or participate in research unless referred by trusted peers, the initial “known individuals” from whom the snowball sampling began. No ex-post filtering was applied.

### Data collection tool

We chose to collect data online to facilitate access, accommodating physicians’ busy schedules and ensuring participants felt comfortable and unpressured when responding. The questionnaire was distributed as a digital Google Form and sent to participants via various social networks, including Facebook, Twitter, WhatsApp, and others. The estimated completion time was approximately 10–12 minutes. The questionnaire comprised four sections (see “S1 Appendix”).

A General characteristics of the studied population: age, sex, current marital status, and highest academic qualification.
B Work-related information: medical specialty, current clinical position, primary work shift, workplace location, type of healthcare facility, employment type, years of experience in the current specialty, and average number of patients seen per day.
C Questions to assess the medico-legal claims raised by patients against physicians: frequency, insurance coverage, and their consequences.
D Questions assessing positive (Assurance Behavior) and negative (Avoidance Behavior) DM practices using the Defensive Medicine Behavior Scale (DMBS). The DMBS comprises 14 items: the first nine assess positive DM, and the remaining five assess negative DM. Responses were recorded on a 5-point Likert scale ranging from “1: strongly disagree” to “5: strongly agree.” A total score was calculated for each participant, with a possible range of 14–70. These total scores were categorized as follows: very high (56–70 points), high (42–55 points), moderate (28–41 points), and low (14–27 points) [24].

The usability and technical functionality of the electronic questionnaire were tested by the authors and their colleagues prior to distribution. A feature in Google Forms was used to prevent duplicate submissions by notifying participants when they attempted to resubmit if their responses had already been submitted.

### Ethical considerations

The study was approved by the Research Ethics Committee (REC) of the Faculty of Medicine, Suez Canal University (approval code: 5532). All participants were required to provide informed consent prior to participation using a written consent process at the beginning of the self-administered questionnaire. The questionnaire’s introductory section outlined the study’s purpose, emphasized the voluntary nature of participation, and assured participants of data confidentiality and exclusive use for research purposes. Additionally, responses were collected anonymously, and participant data were coded to ensure confidentiality. No incentives were offered for participation. Participants retained the right to refuse or withdraw from the study at any time without providing a reason and without facing negative consequences. Furthermore, the authors’ contact information was provided for participants to request clarification if needed.

### Data management and statistical analysis

Data were coded and entered using Microsoft Excel 2016. Only fully completed responses were included in the analysis, which was performed using IBM SPSS Statistics, version 22.0. Descriptive statistics were used to summarize participant characteristics and work-related information. Numerical variables are presented as mean and standard deviation, as well as median with range (minimum–maximum), while categorical variables are expressed as frequency and percentage (%). The prevalence of DM was calculated as the percentage of participants who reported practicing any DM behavior on the DMBS. The normality of data distribution was assessed using the Kolmogorov–Smirnov test.

Analytic statistics were applied based on the nature of the data and variables to compare surgical and non-surgical specialties. The Mann-Whitney U test was used to evaluate differences in non-normally distributed numerical data, while the Chi-square test was applied to analyze categorical data. A P-value < 0.05 was considered statistically significant. To identify potential determinants of DM practices, a multivariate logistic regression analysis was performed, with results expressed as odds ratios (OR) and 95% confidence intervals (CI). A bar chart was used to present the prevalence of DM and the differences between the surgical and non-surgical specialties.

## Results

The study enrolled 210 physicians with a mean age of 39 ± 7 years, with a nearly equal sex distribution (1:1). Most participants were married (79%), and approximately half (51.4%) held the highest qualification of Doctor of Medicine or a Ph.D. in Philosophy (Table 1).

**Table 1:** General characteristics of the participants studied.

General characteristics				
	Mean	SD	Median	Min-Max
**Age (years)**	39	7	38	23-67
Frequency (%)				
**Sex**	Male			104 (49.5)
	Female			106 (50.5)
**Marital status**	Single			35 (16.7)
	Married			166 (79)
	Divorced			8 (3.8)
	Widow			1 (0.5)
**Highest qualification**	Bachelor’s degree in medicine and surgery			24 (11.4)
	Diploma			4 (1.9)
	Master			62 (29.5)
	Egyptian fellowship			12 (5.7)
	M.D. or Ph.D.^†^			108 (51.4)

Quantitative variables are presented as mean ± SD and median (Min-Max), whereas categorical variables are presented as frequency (%).

^†^M.D. Doctor of Medicine, Ph.D. Doctor of Philosophy.

The participants were categorized into two groups: 114 (54.3%) from non-surgical specialties and 96 (45.7%) from surgical specialties. A majority (61.9%) were consultants, and approximately half (51.4%) worked both day and night shifts. Regarding their workplace, most (59%) were employed in urban settings. The most common workplaces were university hospitals, the private sector, and health insurance organizations, which accounted for 60.5%, 40%, and 35.2% of participants, respectively (Table 2).

**Table 2:** Work-related information about the participants studied.

Work-related information			Frequency (%)	
**Specialty**	Surgical		96 (35.7)	
	Non-surgical		114 (54.3)	
**Current clinical position**	Resident		25 (11.9)	
	Specialist		55 (26.2)	
	Consultant		130 (61.9)	
**Main work shift (day/night)**	Day		97 (46.2)	
	Night		5 (2.4)	
	Both		108 (51.4)	
**Workplace location**	Urban		124 (59)	
	Rural		15 (7.1)	
	Both		71 (33.8)	
**Workplace type**	University Hospital		127 (60.5)	
	Health insurance organization		74 (35.2)	
	Military hospital		11 (5.2)	
	Ministry of Health hospital/health center		38 (18.1)	
	Private sector		84 (40)	
	Primary health care unit		36 (17.1)	
**Type of employment**	Contract		23 (11)	
	Permanent		100 (47.6)	
	Both contract and permanent		87 (41.4)	
	Mean	SD	Median	Min-Max
**Average time of experience in specialty (years)**	13	7	13	1-40
**Average number of cases usually examined per day**	24	15	20	2-50

Quantitative variables are presented as mean ± SD and median (Min-Max), whereas categorical variables are presented as frequency (%).

Throughout their careers, physicians in surgical specialties faced a higher average number of malpractice accusations than those in non-surgical specialties. Surgical specialists, however, had significantly lower workplace insurance coverage for such medico-legal claims (P-value <0.05) (Table 3).

**Table 3:** Medico-legal claims against the participants studied.

Medico-legal claims		Surgical (n = 96)	Non-surgical (n = 114)	P-value
**Average number of claims raised against a physician**	Mean±SD	11 ± 17	6 ± 10	0.022^*^
	Median (min-max)	4 (0-50)	2 (0-50)	
**Workplace offering insurance for medico-legal claims** ^§^	Covered	18 (18.8)	23 (20.2)	0.002^*^
	Not covered	54 (56.3)	39 (34.2)	
	Don’t know	24 (25)	52 (45.6)	

Quantitative variables are presented as mean ± SD and median (Min-Max), whereas categorical variables are presented as frequency (%).

* P-value is statistically significant.

Regarding physician concerns following an accusation, both groups reported high levels of concern across multiple categories. However, only the concern about “loss of reputation among colleagues” was reported significantly more frequently by physicians in non-surgical specialties (P-value <0.05) (Table 4).

**Table 4:** Sequences that cause concern for physicians in the event of a medico-legal claim.

Sequences cause concern for physicians ^§^	Surgical (n = 96)	Non-surgical (n = 114)	P-value
Blame from colleagues	38 (39.6)	50 (43.9)	0.576
Disciplinary action by a professional body	53 (55.2)	69 (60.5)	0.484
Financial impact	55 (57.3)	60 (52.6)	0.578
Loss of reputation among colleagues	60 (62.5)	87 (76.3)	0.035^*^
Malpractice litigation	76 (79.2)	86 (75.4)	0.621
Negative patient or family reaction	75 (78.1)	85 (74.6)	0.626
Negative publicity from the news media	61 (63.5)	78 (68.4)	0.468

^§^Frequency of yes responses (%).

* P-value is statistically significant.

**Fig 1:**
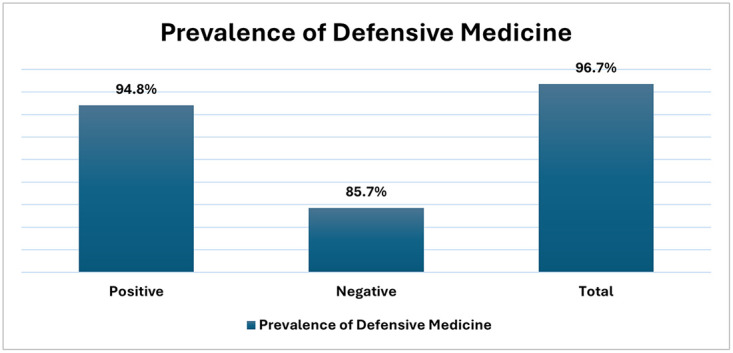
The prevalence of defensive medicine among the studied participants.

The prevalence of DM was 94.8% for positive practices, 85.7% for negative practices, and 96.7% overall (Fig 1). Physicians in surgical specialties reported engaging in positive DM practices more frequently than those in non-surgical specialties, specifically in behaviors such as “ordering additional tests for legal protection,” “utilizing imaging techniques more often to avoid legal issues,” and “emphasizing informed consent forms to protect themselves legally” (P-value <0.05). In contrast, there were no statistically significant differences between specialties in the practice of negative DM. Furthermore, the overall prevalence rates of positive, negative, and total DM practices showed no statistically significant difference between the two groups (Table 5).

**Table 5:** Practice of defensive medicine among the participants studied.

Positive defensive medicine practices^§^	Surgical (n = 96)	Non-surgical (n = 114)	P-value
**1.** I order extra tests for my patients for legal protection	50 (52.1)	42 (36.8)	0.036^*^
**2.** I hospitalize patients for reasons other than indications (e.g., social indication) in order to avoid legal problems	17 (17.7)	16 (14)	0.569
**3.** I prescribe as many drugs as I can in order to avoid legal problems	7 (7.3)	10 (8.8)	0.802
**4.** I spend more time with my patients in order to protect myself legally	46 (47.9)	58 (50.9)	0.68
**5.** I explain medical procedures to my patients in more detail in order to protect myself legally	85 (88.5)	102 (89.5)	1
**6.** I order more consultations on possible complications in order to avoid legal problems	73 (76)	75 (65.8)	0.129
**7.** I use imaging techniques more often in order to avoid legal problems	59 (61.5)	46 (40.4)	0.004^*^
**8.** I keep more detailed records in order to avoid legal problems	73 (76)	76 (66.7)	0.17
**9.** I place more emphasis on informed consent forms in order to protect myself legally	75 (78.1)	70 (61.4)	0.011^*^
**Prevalence of positive defensive medicine practices** ^§^	93 (96.6)	106 (93)	0.233
**Negative defensive medicine practices** ^§^			
1. I prefer to use non-invasive protocols instead of interventional treatment protocols in order to avoid legal problems	49 (51)	69 (60.5)	0.209
2. I avoid treatment protocols with high complication rates in order to avoid problems	52 (54.2)	67 (58.8)	0.576
3. I avoid patients with complex medical problems in order to avoid legal problems	39 (40.6)	38 (33.3)	0.315
4. I avoid patients who are likely to sue in order to avoid legal problems	49 (51)	52 (45.6)	0.489
5. I feel uncomfortable in practice as malpractice is appearing more frequently in the media	54 (56.3)	64 (56.1)	1
**Prevalence of negative defensive medicine practices** ^§^	82 (85.4)	98 (86)	1
**The total prevalence of defensive medicine practices** ^§^	94 (97.9)	109 (95.6)	0.458

^§^Frequency (%).

* P-value is statistically significant.

**Fig 2:**
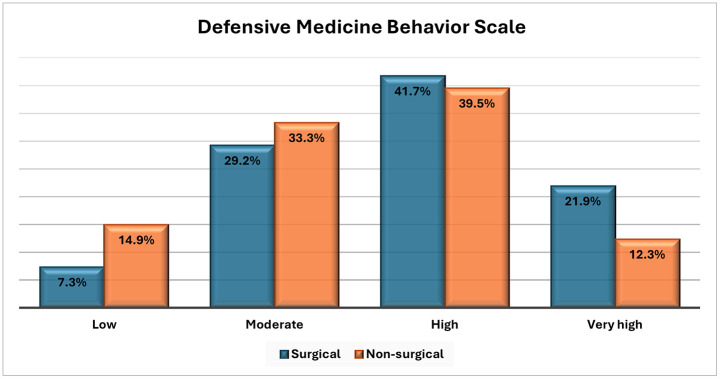
The practice of defensive medicine between surgical and non-surgical specialties.

Overall, a high level of DM practice was observed among physicians in both surgical and non-surgical specialties, with prevalences of 41.7% and 39.5%, respectively. However, this difference between the two groups was not statistically significant (P-value > 0.05) (Fig 2).

The regression analysis assessing factors associated with the practice of positive DM began with an unadjusted parsimonious model, which indicated that specialty type was a statistically significant predictor (Table 6). After adjusting for general characteristics—including age, sex, marital status, and the physician’s highest qualification—specialty type became non-significant, suggesting that the inclusion of additional independent variables influenced the outcome. In this adjusted model, male sex and holding only a bachelor’s degree were also identified as statistically significant predictors (Table 7).

**Table 6:** Univariate regression analysis of practicing positive defensive medicine.

Independent variables	Unstandardized coefficients		Standardized coefficients	t	Sig.	95.0% Confidence interval for B	
	B	Std. Error	Beta			Lower bound	Upper bound
Specialty	−.710	.289	−.168	−2.453	.015*	−1.281	−.139

* P-value is statistically significant.

**Table 7:** Multivariate regression analysis of practicing positive defensive medicine after adjusting for general characteristics.

Independent variables	Unstandardized coefficients		Standardized coefficients	t	Sig.	95.0% Confidence interval for B	
	B	Std. Error	Beta			Lower bound	Upper bound
Specialty	−.363	.297	−.086	−1.221	.223	−.949	.223
Age	−.023	.022	−.079	−1.034	.303	−.066	.021
Sex	.809	.304	.192	2.658	.008*	.209	1.409
Marital status	−.135	.131	−.073	−1.031	.304	−.392	.123
Highest qualification	−.250	.117	−.163	−2.137	.034*	−.481	−.019

* P-value is statistically significant.

Subsequent adjustment for work-related information (Table 8) showed that male sex, holding only a bachelor’s degree, and being a consultant were statistically significant predictors. Finally, adding data on medico-legal claims raised against the participants (Table 9) revealed that working at university hospitals (β = −0.178) and having workplace insurance for medico-legal claims (β = −0.153) were statistically associated with fewer positive DM practices. Conversely, concerns about the financial implications of medico-legal claims (β = 0.26) and negative reactions from patients or families (β = 0.157) were statistically associated with more positive DM practices (P-value < 0.05).

**Table 8:** Multivariate regression analysis of practicing positive defensive medicine after adjusting for general characteristics and work-related information.

Independent variables	Unstandardized coefficients		Standardized Coefficients	t	Sig.	95.0% Confidence interval for B	
	B	Std. Error	Beta			Lower bound	Upper bound
Specialty	−.438	.331	−.103	−1.324	.187	−1.090	.214
Age	−.002	.065	−.005	−.024	.981	−.129	.126
Sex	.712	.338	.169	2.104	.037*	.044	1.379
Marital status	−.089	.142	−.049	−.628	.531	−.369	.191
Highest qualification	−.569	.256	−.370	−2.226	.027*	−1.073	−.065
Current Clinical Position	1.139	.552	.377	2.063	.040*	.050	2.228
Main Work Shift (Day/Night)	−.005	.169	−.002	−.028	.977	−.338	.328
Workplace location	.021	.162	.009	.126	.900	−.299	.340
University hospital	−.618	.371	−.143	−1.665	.098	−1.350	.114
Health insurance organization	−.353	.326	−.080	−1.084	.280	−.996	.290
Military hospital	.107	.654	.011	.164	.870	−1.184	1.398
Ministry of Health hospital/health centre	−.314	.388	−.057	−.809	.420	−1.079	.452
Private sector	−.086	.326	−.020	−.264	.792	−.730	.558
Primary health care unit	−.824	.426	−.147	−1.935	.054	−1.664	.016
Type of employment	.192	.253	.060	.757	.450	−.308	.691
Average time of experience in the specialty	−.064	.074	−.216	−.870	.385	−.211	.082
The average number of cases usually examined per day	.004	.010	.025	.358	.721	−.016	.023

* P-value is statistically significant.

**Table 9:** Multivariate regression analysis of practicing positive defensive medicine after adjusting for general characteristics, work-related information, and medico-legal claims raised against the studied participants.

Independent variables	Unstandardized coefficients		Standardized coefficients	t	Sig.	95.0% Confidence interval for B	
	B	Std. Error	Beta			Lower bound	Upper bound
Specialty	−.452	.325	−.107	−1.392	.166	−1.092	.189
Age	−.012	.061	−.043	−.202	.841	−.133	.108
Sex	.546	.325	.129	1.681	.094	−.095	1.186
Marital status	−.153	.135	−.084	−1.134	.258	−.420	.114
Highest qualification	−.411	.244	−.267	−1.684	.094	−.892	.070
Current Clinical Position	.517	.537	.171	.963	.337	−.542	1.576
Main Work Shift (Day/Night)	−.058	.162	−.027	−.362	.718	−.377	.260
Workplace location	.038	.155	.017	.244	.808	−.269	.345
University hospital	−.767	.353	−.178	−2.173	.031*	−1.464	−.071
Health insurance organization	−.141	.316	−.032	−.446	.656	−.765	.483
Military hospital	−.166	.623	−.018	−.267	.790	−1.395	1.063
Ministry of Health hospital/health centre	−.424	.369	−.077	−1.149	.252	−1.153	.304
Private sector	−.092	.311	−.021	−.295	.769	−.704	.521
Primary health care unit	−.728	.410	−.130	−1.776	.077	−1.538	.081
Type of employment	.316	.241	.098	1.308	.192	−.160	.792
Average time of experience in the specialty	−.017	.071	−.058	−.247	.805	−.157	.122
The average number of cases usually examined per day	.002	.010	.016	.240	.811	−.017	.021
Average number of claims raised against physician	−.005	.010	−.033	−.511	.610	−.025	.015
Workplace offering insurance for medico-legal claims	−.444	.195	−.153	−2.278	.024*	−.828	−.059
Blame from colleagues	.192	.298	.045	.643	.521	−.397	.781
Disciplinary action by a professional body	.224	.320	.052	.700	.485	−.407	.855
Financial impact	1.100	.323	.260	3.403	.001*	.462	1.738
Loss of reputation among colleagues	.392	.331	.085	1.187	.237	−.260	1.045
Malpractice litigation	.453	.345	.090	1.313	.191	−.227	1.133
Negative patient or family reaction	.777	.344	.157	2.260	.025*	.099	1.455
Negative publicity from the news media	−.127	.322	−.029	−.395	.694	−.763	.509

* P-value is statistically significant.

In contrast, the unadjusted parsimonious model evaluating the predictive role of specialty type for negative DM showed no statistical significance (Table 10). This remained the case after sequentially adjusting for the physicians’ general characteristics (age, sex, marital status, and highest qualification; Table 11), work-related information (Table 12), and medico-legal claims against participants (Table 13). No statistically significant predictors of negative DM were identified in any of these adjusted models.

**Table 10:** Univariate regression analysis of practicing negative defensive medicine.

Independent variables	Unstandardized coefficients		Standardized coefficients	t	Sig.	95.0% Confidence interval for B	
	B	Std. Error	Beta			Lower bound	Upper bound
Specialty	.013	.232	.004	.054	.957	−.445	.470

**Table 11:** Multivariate regression analysis of practicing negative defensive medicine after adjusting for general characteristics.

Independent variables	Unstandardized coefficients		Standardized coefficients	t	Sig.	95.0% Confidence interval for B	
	B	Std. Error	Beta			Lower bound	Upper bound
Specialty	.163	.244	.049	.667	.506	−.319	.645
Age	−.009	.018	−.041	−.518	.605	−.045	.026
Sex	.346	.250	.104	1.382	.168	−.148	.840
Marital status	−.176	.107	−.121	−1.641	.102	−.388	.035
Highest qualification	−.129	.096	−.106	−1.334	.184	−.319	.061

**Table 12:** Multivariate regression analysis of practicing negative defensive medicine after adjusting the general characteristics and work-related information.

Independent variables	Unstandardized coefficients		Standardized coefficients	t	Sig.	95.0% Confidence interval for B	
	B	Std. Error	Beta			Lower bound	Upper bound
Specialty	.170	.274	.051	.620	.536	−.371	.711
Age	−.004	.054	−.017	−.074	.941	−.109	.102
Sex	.245	.281	.074	.875	.383	−.308	.799
Marital status	−.159	.118	−.109	−1.350	.179	−.391	.073
Highest qualification	−.409	.212	−.337	−1.931	.055	−.827	.009
Current Clinical Position	.850	.458	.356	1.856	.065	−.053	1.753
Main Work Shift (Day/Night)	−.025	.140	−.015	−.177	.860	−.301	.252
Workplace location	.217	.135	.121	1.611	.109	−.049	.482
University hospital	−.111	.308	−.033	−.361	.718	−.718	.496
Health insurance organization	−.096	.270	−.028	−.356	.722	−.629	.437
Military hospital	−.312	.543	−.042	−.575	.566	−1.383	.759
Ministry of Health hospital/health centre	−.046	.322	−.011	−.143	.886	−.681	.589
Private sector	.372	.271	.109	1.375	.171	−.162	.906
Primary health care unit	−.225	.353	−.051	−.637	.525	−.922	.472
Type of employment	−.200	.210	−.079	−.954	.341	−.614	.214
Average time of experience in the specialty	−.037	.061	−.159	−.610	.543	−.159	.084
The average number of cases usually examined per day	−.001	.008	−.012	−.170	.865	−.018	.015

**Table 13:** Multivariate regression analysis of practicing negative defensive medicine after adjusting the general characteristics, work-related information, and medico-legal claims raised against the studied participants.

Independent variables	Unstandardized coefficients		Standardized coefficients	t	Sig.	95.0% Confidence interval for B	
	B	Std. Error	Beta			Lower bound	Upper bound
Specialty	.078	.279	.023	.280	.780	−.473	.629
Age	.001	.053	.006	.024	.981	−.102	.105
Sex	.090	.279	.027	.323	.747	−.461	.641
Marital status	−.186	.116	−.128	−1.600	.111	−.416	.043
Highest qualification	−.316	.210	−.260	−1.508	.133	−.730	.098
Current Clinical Position	.527	.462	.221	1.142	.255	−.384	1.439
Main Work Shift (Day/Night)	−.044	.139	−.026	−.317	.751	−.318	.230
Workplace location	.167	.134	.093	1.250	.213	−.097	.431
University hospital	−.238	.304	−.070	−.784	.434	−.838	.361
Health insurance organization	−.052	.272	−.015	−.191	.849	−.589	.485
Military hospital	−.385	.536	−.051	−.719	.473	−1.443	.672
Ministry of Health hospital/health centre	−.126	.318	−.029	−.397	.692	−.753	.501
Private sector	.399	.267	.117	1.494	.137	−.128	.926
Primary health care unit	−.173	.353	−.039	−.490	.625	−.869	.523
Type of employment	−.201	.208	−.079	−.969	.334	−.611	.209
Average time of experience in the specialty	−.032	.061	−.137	−.534	.594	−.152	.087
The average number of cases usually examined per day	.000	.008	−.004	−.058	.954	−.017	.016
Average number of claims raised against physician	.007	.009	.060	.843	.400	−.010	.024
Workplace offering insurance for medico-legal claims	−.046	.168	−.020	−.272	.786	−.376	.285
Blame from colleagues	.242	.257	.072	.943	.347	−.264	.748
Disciplinary action by a professional body	−.170	.275	−.050	−.618	.537	−.713	.373
Financial impact	.545	.278	.163	1.959	.052	−.004	1.094
Loss of reputation among colleagues	.418	.284	.115	1.470	.143	−.143	.979
Malpractice litigation	−.156	.297	−.039	−.526	.600	−.741	.429
Negative patient or family reaction	.230	.296	.059	.778	.437	−.353	.813
Negative publicity from the news media	.526	.277	.149	1.896	.060	−.021	1.073

Multivariable-adjusted regression analyses were conducted separately for surgical and non-surgical specialties (Tables 14–17). The results showed that, among surgical specialists, only concerns about the financial implications of medico-legal claims (β = 0.299) were significantly associated with a greater likelihood of positive DM practices (P < 0.05). Among non-surgical specialists, however, working in the private sector (β = 0.27) and a higher average number of malpractice claims filed against the physician (β = 0.202) were significantly associated with more frequent negative DM practices (P < 0.05).

**Table 14:** Multivariate regression analysis of practicing positive defensive medicine among physicians in surgical specialties, adjusted for general characteristics, work-related information, and medico-legal claims.

Independent variables	Unstandardized coefficients		Standardized coefficients	t	Sig.	95.0% Confidence interval for B	
	B	Std. Error	Beta			Lower bound	Upper bound
Age	.073	.143	.249	.511	.611	−.212	.359
Sex	.281	.579	.065	.485	.629	−.875	1.437
Marital status	−.121	.252	−.069	−.481	.632	−.623	.381
Highest qualification	−.813	.510	−.588	−1.595	.115	−1.830	.204
Current Clinical Position	1.841	1.110	.672	1.660	.101	−.372	4.054
Main Work Shift (Day/Night)	−.327	.279	−.142	−1.169	.246	−.884	.230
Workplace location	.147	.265	.068	.553	.582	−.382	.675
University hospital	−.857	.666	−.192	−1.287	.202	−2.184	.471
Health insurance organization	−.223	.511	−.053	−.436	.664	−1.242	.796
Military hospital	.649	1.104	.071	.588	.559	−1.553	2.851
Ministry of Health hospital/health centre	−.520	.557	−.101	−.933	.354	−1.632	.592
Private sector	.242	.516	.059	.469	.640	−.787	1.271
Primary health care unit	−.736	1.160	−.072	−.634	.528	−3.050	1.579
Type of employment	.184	.406	.060	.452	.653	−.627	.994
Average time of experience in the specialty	−.147	.152	−.522	−.970	.335	−.450	.156
The average number of cases usually examined per day	.002	.016	.013	.107	.915	−.030	.033
Average number of claims raised against physician	−.002	.014	−.015	−.133	.895	−.029	.025
Workplace offering insurance for medico-legal claims	−.504	.357	−.163	−1.412	.162	−1.216	.208
Blame from colleagues	.368	.508	.088	.725	.471	−.645	1.382
Disciplinary action by a professional body	.110	.546	.027	.201	.841	−.979	1.199
Financial impact	1.234	.540	.299	2.285	.025*	.157	2.310
Loss of reputation among colleagues	.351	.522	.083	.673	.503	−.689	1.391
Malpractice litigation	.462	.589	.092	.785	.435	−.712	1.637
Negative patient or family reaction	.573	.607	.116	.944	.348	−.638	1.785
Negative publicity from the news media	−.078	.519	−.018	−.151	.880	−1.113	.956

* P-value is statistically significant.

**Table 15:** Multivariate regression analysis of practicing negative defensive medicine after adjusting for general characteristics, work-related information, and medico-legal claims among physicians in surgical specialties.

Independent variables	Unstandardized coefficients		Standardized coefficients	t	Sig.	95.0% Confidence interval for B	
	B	Std. Error	Beta			Lower bound	Upper bound
Age	.035	.128	.143	.272	.787	−.221	.291
Sex	.055	.519	.015	.106	.916	−.981	1.090
Marital status	−.117	.226	−.080	−.517	.607	−.567	.333
Highest qualification	−.408	.457	−.355	−.892	.375	−1.319	.503
Current Clinical Position	1.022	.994	.449	1.028	.307	−.961	3.005
Main Work Shift (Day/Night)	−.121	.250	−.063	−.485	.629	−.621	.378
Workplace location	.220	.238	.124	.928	.357	−.253	.694
University hospital	.105	.596	.028	.176	.861	−1.084	1.295
Health insurance organization	.416	.458	.120	.907	.367	−.498	1.329
Military hospital	.053	.989	.007	.054	.957	−1.920	2.027
Ministry of Health hospital/health centre	−.089	.500	−.021	−.177	.860	−1.085	.908
Private sector	−.050	.462	−.015	−.108	.914	−.972	.872
Primary health care unit	.203	1.040	.024	.195	.846	−1.871	2.276
Type of employment	−.299	.364	−.118	−.820	.415	−1.025	.428
Average time of experience in the specialty	−.049	.136	−.208	−.358	.722	−.320	.223
The average number of cases usually examined per day	−.001	.014	−.011	−.085	.933	−.029	.027
Average number of claims raised against physician	−.005	.012	−.052	−.441	.661	−.030	.019
Workplace offering insurance for medico-legal claims	.119	.320	.046	.373	.711	−.519	.757
Blame from colleagues	.471	.456	.136	1.033	.305	−.438	1.379
Disciplinary action by a professional body	.162	.489	.047	.331	.742	−.814	1.138
Financial impact	.403	.484	.118	.834	.407	−.561	1.368
Loss of reputation among colleagues	.511	.467	.146	1.094	.278	−.421	1.443
Malpractice litigation	−.055	.528	−.013	−.105	.917	−1.108	.997
Negative patient or family reaction	.012	.544	.003	.022	.982	−1.073	1.097
Negative publicity from the news media	.703	.465	.200	1.513	.135	−.224	1.631

**Table 16:** Multivariate regression analysis of practicing positive defensive medicine among physicians in non-surgical specialties, adjusted for general characteristics, work-related information, and medico-legal claims.

Independent variables	Unstandardized coefficients		Standardized coefficients	t	Sig.	95.0% Confidence interval for B	
	B	Std. Error	Beta			Lower bound	Upper bound
Age	−.057	.076	−.205	−.748	.456	−.208	.094
Sex	.606	.484	.137	1.252	.214	−.356	1.567
Marital status	−.235	.202	−.126	−1.162	.249	−.637	.167
Highest qualification	−.403	.337	−.243	−1.198	.234	−1.073	.266
Current Clinical Position	−.100	.748	−.031	−.133	.894	−1.587	1.388
Main Work Shift (Day/Night)	.146	.237	.066	.616	.539	−.324	.616
Workplace location	−.123	.240	−.053	−.510	.611	−.600	.355
University hospital	−.345	.510	−.082	−.676	.501	−1.359	.669
Health insurance organization	−.046	.508	−.010	−.090	.929	−1.055	.964
Military hospital	−.330	.948	−.035	−.348	.729	−2.214	1.555
Ministry of Health hospital/health centre	−.104	.593	−.018	−.175	.862	−1.281	1.074
Private sector	−.433	.465	−.096	−.931	.354	−1.356	.491
Primary health care unit	−.622	.520	−.132	−1.197	.235	−1.654	.411
Type of employment	.384	.387	.117	.993	.324	−.385	1.154
Average time of experience in the specialty	.074	.097	.241	.763	.447	−.118	.266
The average number of cases usually examined per day	.009	.016	.057	.536	.593	−.024	.041
Average number of claims raised against physician	−.005	.020	−.026	−.266	.791	−.046	.035
Workplace offering insurance for medico-legal claims	−.456	.290	−.167	−1.573	.119	−1.033	.120
Blame from colleagues	.169	.449	.040	.377	.707	−.723	1.062
Disciplinary action by a professional body	.458	.459	.106	.997	.321	−.455	1.371
Financial impact	.869	.491	.205	1.769	.080	−.107	1.844
Loss of reputation among colleagues	.377	.525	.076	.718	.474	−.666	1.421
Malpractice litigation	.701	.534	.143	1.313	.192	−.360	1.761
Negative patient or family reaction	.770	.495	.159	1.556	.123	−.213	1.752
Negative publicity from the news media	−.310	.499	−.068	−.620	.537	−1.302	.683

**Table 17:** Multivariate regression analysis of negative defensive medicine practice after adjusting for general characteristics, work-related information, and medico-legal claims among physicians in non-surgical specialties.

Independent variables	Unstandardized coefficients		Standardized coefficients	t	Sig.	95.0% Confidence interval for B	
	B	Std. Error	Beta			Lower bound	Upper bound
Age	.003	.060	.016	.056	.955	−.116	.122
Sex	.244	.381	.071	.642	.523	−.513	1.001
Marital status	−.192	.159	−.132	−1.205	.231	−.509	.125
Highest qualification	−.206	.265	−.159	−.778	.439	−.733	.321
Current Clinical Position	.040	.589	.016	.069	.945	−1.131	1.212
Main Work Shift (Day/Night)	−.062	.186	−.036	−.332	.741	−.432	.308
Workplace location	.126	.189	.070	.665	.508	−.250	.502
University hospital	−.489	.402	−.149	−1.217	.227	−1.288	.309
Health insurance organization	−.407	.400	−.115	−1.017	.312	−1.201	.388
Military hospital	−.222	.747	−.030	−.297	.767	−1.705	1.262
Ministry of Health hospital/health centre	−.341	.467	−.077	−.731	.467	−1.269	.586
Private sector	.947	.366	.270	2.587	.011*	.220	1.674
Primary health care unit	−.026	.409	−.007	−.064	.949	−.839	.787
Type of employment	−.320	.305	−.125	−1.050	.296	−.926	.286
Average time of experience in the specialty	−.051	.076	−.212	−.663	.509	−.202	.101
The average number of cases usually examined per day	.005	.013	.038	.358	.722	−.021	.030
Average number of claims raised against physician	.032	.016	.202	2.017	.047*	.000	.064
Workplace offering insurance for medico-legal claims	−.249	.228	−.117	−1.092	.278	−.703	.205
Blame from colleagues	.042	.354	.013	.118	.906	−.661	.744
Disciplinary action by a professional body	−.363	.362	−.108	−1.003	.319	−1.082	.356
Financial impact	.677	.387	.205	1.750	.084	−.092	1.445
Loss of reputation among colleagues	.460	.414	.119	1.113	.269	−.362	1.282
Malpractice litigation	−.103	.420	−.027	−.245	.807	−.938	.732
Negative patient or family reaction	.489	.389	.130	1.256	.212	−.285	1.263
Negative publicity from the news media	.529	.393	.150	1.345	.182	−.252	1.310

* P-value is statistically significant.

## Discussion

The present study offers an in-depth analysis of DM practices among physicians in both surgical and non-surgical specialties. The findings demonstrate that DM is widespread, with a substantial proportion of physicians in both groups engaging in such practices, particularly in the form of positive defensive strategies. Despite this high prevalence, no statistically significant difference in overall engagement was observed between the surgical and non-surgical groups.

These findings are consistent with a similar study in Egypt, which reported that DM is deeply ingrained in the clinical routines of Egyptian physicians [25]. Likewise, a previous study conducted among Egyptian residents at Kasr Alainy Hospital found a high prevalence of DM practices and associated feelings of insecurity [23].

Similarly, a study at Tanta University Hospitals in Egypt during the COVID-19 pandemic revealed a highly evident prevalence of DM, primarily driven by physicians’ legal self-interest [19]. Furthermore, a comparative study of DM practices between Egypt and Saudi Arabia reported that Egyptian physicians engaged in these behaviors more frequently than their Saudi counterparts [20].

Globally, a cross-sectional study involving 220 physicians at a university hospital in Turkey found that an overwhelming majority (94.2%) reported engaging in some form of DM [14]. Another study assessing DM among surgeons in Ethiopia found that a majority (74%) engaged in such practices, most commonly avoiding high-risk procedures and ordering unnecessary tests [26]. Furthermore, a scoping review revealed that the prevalence of DM among physicians ranges widely, from 6.7% to 99.8% [13]. A recent systematic review and meta-analysis further reported a pooled prevalence of 75.8% [27].

In the current study, surgeons reported practicing positive DM, including behaviors such as ordering extra tests for legal protection, using imaging techniques more frequently to avoid legal problems, and placing greater emphasis on informed consent forms for legal self-protection. This increased engagement in positive defensive practices may be explained by the higher incidence of medico-legal claims against surgeons and their relatively lower workplace coverage for medico-legal insurance.

This finding aligns with the recent systematic review by Ries and Jansen [28] of empirical research on physicians’ views and experiences of DM. Their review found that the provision of unnecessary services to mitigate perceived legal risk, a hallmark of DM, is frequently reported, particularly in the United States, Europe, and within surgical and obstetrical fields. This pattern may be explained by the higher frequency of damage claims and the greater financial burdens associated with claims in surgical specialties such as general surgery, orthopedics, and gynecology, compared to non-surgical specialties [29].

Interestingly, the study found that non-surgical specialists were more concerned about losing their reputation among colleagues. This may be because patient trust when selecting a non-surgical specialist depends heavily on professional reputation. In contrast, for surgical specialists, a patient’s choice is dominated more by the physician’s technical skills and procedural expertise [30,31].

The regression analysis showed that working at university hospitals and having workplace insurance coverage for medico-legal claims were associated with reduced positive DM practices. This may be explained by the fact that large, hierarchically structured healthcare institutions [32], along with those providing reasonable medico-legal insurance coverage, likely play an important role in mitigating physicians’ defensive practices [9].

On the other hand, concerns regarding the financial implications of medico-legal claims and about negative reactions from patients or their families were associated with increased positive DM practices. This may be attributed to the fact that some medico-legal claims result in physicians paying substantial compensation out-of-pocket, particularly in the absence of insurance coverage. This financial risk, in turn, incentivizes physicians to practice more defensively, as ordering additional tests or imaging may protect them from future financial liability [8]. Furthermore, negative reactions from patients or families can seriously damage a physician’s reputation, potentially making them less preferred by future patients [30].

This study represents a significant initial step toward elucidating critical associations within the Egyptian context, while acknowledging that DM is a multifactorial phenomenon.

### Limitations

The findings of this study should be interpreted within the context of several limitations. The primary limitation is the cross-sectional design; while appropriate for the study’s objectives, it does not allow for the determination of a temporal relationship between medical specialty and the practice of DM. Additionally, the relatively small sample size, the potential for volunteer bias, and the lack of random sampling may limit the generalizability of the findings. Furthermore, the binary categorization of specialties into “surgical” and “non-surgical” groups is an oversimplification that could introduce dilution bias, as many specialties incorporate both procedural and non-procedural elements to varying degrees. Nevertheless, this classification allows for a meaningful comparison between physicians who primarily work in high-risk, procedural environments (surgical) and those whose practice is largely non-operative (non-surgical), despite some inherent overlap.

Moreover, although this study examined the effect of many factors on defensive medicine, other potential determinants, were not assessed including organizational culture, workload, patient expectations, and institutional support, were not fully assessed. This limitation highlights the complexity of defensive medicine and indicates the need for future research better understand the effect of these additional contributing factors.

### Conclusion

Despite the high prevalence of defensive medicine (DM) practices, no statistically significant difference was observed between surgical and non-surgical groups in their overall engagement in these behaviors. Working in university hospitals and having workplace insurance coverage for medico-legal claims were associated with fewer positive DM practices. In contrast, concerns about the financial implications of medico-legal claims and about negative reactions from patients or their families were associated with a greater prevalence of positive DM practices. Therefore, curtailing these practices, specifically by reducing unnecessary healthcare services that expose patients to additional risk, could improve clinical decision-making and care quality. Moreover, reducing DM would lower overall healthcare costs and allow resources to be reallocated toward more value-based care, thereby enhancing cost-effectiveness within the healthcare system.

### Recommendations

The high prevalence of defensive practices across specialties underscores the need for systemic changes to address root causes, such as the fear of litigation, rather than focusing on specialty-specific interventions. The legal framework and malpractice laws require reassessment by the Egyptian Parliament to establish a more balanced system that protects both patients’ rights and physicians’ professional integrity. Such legal reform could reduce the perceived need for physicians to practice DM. The General Authority for Health Insurance (GAHI) and private insurance companies offering professional liability coverage should also expand medico-legal insurance coverage for physicians across all specialties. Furthermore, this study recommends incorporating medico-legal training and patient safety education into medical curricula to improve physicians’ understanding of their legal responsibilities and risks, which may reduce their reliance on defensive practices. Finally, further research is needed to investigate other factors influencing DM, in order to capture the full complexity of this phenomenon, including but not limited to organizational culture, workload, patient expectations, and institutional support.

## Supporting information

S1 Appendix This appendix lists all the questions included in the data collection tool used for the study. (DOCX)

S1 Dataset Dataset. (XLSX)
