# Supplementary material for: Comparison of accusations against physicians and the practice of defensive medicine between surgical and non-surgical specialties
Source: PLoS One. 2026 Mar 5;21(3):e0343807. doi: 10.1371/journal.pone.0343807 (PMC12962507; doi:10.1371/journal.pone.0343807)
Supplement: S1 Appendix — (DOCX) [file pone.0343807.s001.docx]

**S1 Appendix**

**Questionnaire**

**Section (1): General characteristics**

1. **What is your age (in years)?** ……………………….
2. **What is your sex?** m/f
3. **What is your** **current marital status?**

- Married
- Single
- Divorced
- Widow

1. **What is your highest** **academic qualification?**

- Bachelor’s degree in medicine and surgery
- Diploma
- Master
- Egyptian fellowship
- M.D. Doctor of Medicine, Ph.D. Doctor of Philosophy.

**Section (2): Work-related information**

1. **What is your** **medical specialty?**

- Surgical: (e.g., General Surgery, Cardiothoracic Surgery, Plastic and Reconstructive Surgery, Vascular Surgery, Pediatric Surgery, Colorectal Surgery, Oral and Maxillofacial Surgery, Orthopedics, Obstetrics and Gynecology, Orthopedics, Neurosurgery, Otorhinolaryngology, Urology, Ophthalmology, etc.)
- Non-surgical: (e.g., Internal medicine, cardiology, critical care, geriatric, oncology, Allergy and Immunology, Hematology, nephrology, Endocrinology, Gastroenterology, Pulmonology, psychiatry, infectious diseases, Neurology, rheumatology, radiology, Dermatology, Pediatrics, Emergency Medicine, Family Medicine, Geriatric Medicine, Occupational Medicine, Rehabilitation Medicine, Anesthesiology, etc.)

1. **What is your** **current clinical position?**

- Resident
- Specialist
- Consultant

1. **What is your** **main work shift?**

- Day
- Night
- Both

1. **Where is your** **workplace located?**

- Urban
- Rural
- Both

1. **What** **type of healthcare facility do you primarily work in?**

- University hospital
- Health insurance organization
- Military hospital
- Ministry of health hospital/health center
- Private sector
- Primary health care unit

1. **What is your** **type of employment?**

- Contract
- Permanent
- Both contract and permanent

1. **How many years of** **experience do you have in your** **current specialty? …………**
2. **On average, how many patients or cases do you examine per day? ………………**

**Section (3):** **Medico-legal claims against the physician**

1. **How many medical liability (medico-legal) claims have been filed against you during your professional experience? …………………….**
2. **Does your current workplace provide medical liability (malpractice) insurance coverage for physicians?**

- Covered
- Not covered
- Don’t know

1. **Which of the following consequences concern you the most in the event of a medico-legal (malpractice) claim?**

| **Consequences:** | **Yes** | **No** |
| --- | --- | --- |
| Blame from colleagues |  |  |
| Disciplinary action by a professional body |  |  |
| Financial impact |  |  |
| Loss of reputation among colleagues |  |  |
| Malpractice litigation |  |  |
| Negative patient or family reaction |  |  |
| Negative publicity from news media |  |  |

**Section (4): Defensive Medicine Behavior Scale (DMBS)**

| **Defensive Medicine Behaviors:** | **Strongly disagree** | **Disagree** | **Neutral** | **Agree** | **Strongly agree** |
| --- | --- | --- | --- | --- | --- |
| **A. Assurance (Positive Defensive Medicine) Behaviors:** |  |  |  |  |  |
| 1. I order extra tests for my patients for legal protection |  |  |  |  |  |
| 1. I hospitalize patients for reasons other than indications (e.g., social indication) in order to avoid legal problems |  |  |  |  |  |
| 1. I prescribe as many drugs as I can in order to avoid legal problems |  |  |  |  |  |
| 1. I spend more time with my patients in order to protect myself legally |  |  |  |  |  |
| 1. I explain medical procedures to my patients in more detail in order to protect myself legally |  |  |  |  |  |
| 1. I order more consultations on possible complications in order to avoid legal problems |  |  |  |  |  |
| 1. I use imaging techniques more often in order to avoid legal problems |  |  |  |  |  |
| 1. I keep more detailed records in order to avoid legal problems |  |  |  |  |  |
| 1. I place more emphasis on informed consent forms in order to protect myself legally |  |  |  |  |  |
| **B. Avoidance (Negative Defensive Medicine) Behaviors:** |  |  |  |  |  |
| 1. I prefer to use non-invasive protocols instead of interventional treatment protocols in order to avoid legal problems |  |  |  |  |  |
| 1. I avoid treatment protocols with high complication rates in order to avoid problems |  |  |  |  |  |
| 1. I avoid patients with complex medical problems in order to avoid legal problems |  |  |  |  |  |
| 1. I avoid patients who are likely to sue in order to avoid legal problems |  |  |  |  |  |
| 1. I feel uncomfortable in practice as malpractice is appearing more frequently in the media |  |  |  |  |  |
